# Editorial: Role of Lipid Rafts in Anti-microbial Immune Response

**DOI:** 10.3389/fimmu.2021.654776

**Published:** 2021-02-26

**Authors:** Maria Cristina Gagliardi, Kazuhisa Iwabuchi, Chih-Ho Lai

**Affiliations:** ^1^Center for Gender-Specific Medicine, National Institute of Health, Rome, Italy; ^2^Graduate School of Health Care and Nursing, Juntendo University, Urayasu, Japan; ^3^Institute for Environmental and Gender-Specific Medicine, Graduate School of Medicine, Juntendo University, Urayasu, Japan; ^4^Graduate Institute of Biomedical Sciences, Department of Microbiology and Immunology, College of Medicine, Chang Gung University, Taoyuan, Taiwan; ^5^Department of Medical Research, School of Medicine, China Medical University and Hospital, Taichung, Taiwan; ^6^Department of Nursing, Asia University, Taichung, Taiwan; ^7^Department of Pediatrics, Molecular Infectious Disease Research Center, Chang Gung Memorial Hospital, Taoyuan, Taiwan

**Keywords:** lipid rafts, pathogen recognition receptors, microbial phagocytosis, immune response, infection

Lipid rafts are membrane microdomains with heterogeneous and highly dynamic small regions (10–200 nm) rich in sterols and sphingolipids. These unique membrane regions are involved in many crucial biological functions, including the formation of signaling platforms, coalescence of membrane receptors, and amplification of intracellular signaling. Mounting evidence has shown that microbial pathogens, including bacteria, viruses, and parasites, employ these lipid rafts for cellular internalization. Conversely, the engagement of immunological synapses into lipid rafts activates antigen-presenting cells and modulates the T cell response, thus magnifying the host defense against infection. Since pathogens can exploit lipid rafts for cellular interaction via direct contact or virulence factors, disruption or restriction of membrane raft formation can block infection. The five articles presented in this Research Topic collection highlight the current state of knowledge on the interaction of pathogens and their virulence factors with host cell lipid rafts and their roles in infection and disease progression.

Lipid rafts provide membrane platforms for microbial virulence factors and promote their intracellular delivery. Several gram-negative bacteria encode cytolethal distending toxins (Cdts) that contribute to bacterial persistence and invasiveness, which exacerbate disease. These toxins bind to membrane rafts and are internalized and trafficked to target host molecules, resulting in cell cycle arrest, cytokine production, and/or cell toxification. Boesze-Battaglia et al. demonstrate that when macrophages were treated with Cdt encoded by *Aggregatibacter actinomycetemcomitans*, a host cell protein, cellugyrin (synaptogyrin-2), relocates from the detergent-soluble membrane fraction to cholesterol-rich microdomains. Although cellugyrin is not required for Cdt binding to the cell surface, it is essential for its internalization and the induction of pro-inflammatory cytokines. They also demonstrate that Cdt interacts with cellugyrin in a complex that includes Derlin-2, a protein involved in endoplasmic reticulum-associated degradation. Their results provide an important finding that cellugyrin is critical to Cdt translocation and toxicity in human macrophages.

Colonization is an essential initial step in bacterial pathogenesis. Colonization of the host intestinal epithelium and subsequent multiplication in the gut require the contributions of numerous bacterial surface proteins. Chen et al. employ a cell-based model to investigate the role of membrane cholesterol platforms in *Clostridium difficile* infection and show that the binding of surface layer proteins to the host cell membrane efficiently triggers caspase-1-dependent inflammasome activation. Depletion of cellular cholesterol abrogates the inflammasome activation induced by *C. difficile*, indicating that *C. difficile* colonization of the intestinal epithelium is cholesterol dependent. The results of this study may, therefore, answer the clinically relevant question of why statin use reduces the risk of *C. difficile* infection.

*Listeria monocytogenes*, a facultative intracellular pathogen, has been used as a model microorganism to study microbe-host interactions for several decades. Tsai and Chen focus on the role of membrane rafts in the interplay between *Listeria monocytogenes* and host cells. This review summarizes three important issues: (i) the interaction of listeriolysin O with cholesterol-rich microdomains, (ii) the roles of lipid rafts in *Listeria* internalization, and (iii) the roles of lipid rafts in *Listeria* cell-to-cell spread. They emphasize the significance of lipid rafts and raft-associated molecules in the interplay between *L. monocytogenes* and host cells at different interfaces. The review highlights the importance of the recent findings that lipid rafts are involved in the pathophysiology of *L. monocytogenes* infection.

Pathogens hijack membrane rafts to gain access into host cells, and the destruction of raft compartments decreases pathogen infectivity. Severe acute respiratory syndrome coronavirus 2 (SARS-CoV-2) is the etiological agent of coronavirus disease 2019 (COVID-19) and the cause of the ongoing global pandemic since the December of 2019. Accumulating evidence has revealed that reducing the abundance of lipid rafts significantly decreases SARS-CoV-2 infection. The spike protein, located on the viral envelope, binds to angiotensin converting enzyme 2 (ACE2), which is localized to lipid rafts. Thus, ACE2 plays an essential role in initiating viral entry into host cells. Since ACE2 is a raft-associated protein, disruption of the raft architecture by treating cells with methyl-β-cyclodextrin (MβCD) or statins, the most common agents used to disrupt lipid rafts, may be an ideal strategy to reduce SARS-CoV-2 infection. Two reviews of the literature address the issue of “lipid raft therapy” for the treatment of coronavirus infections. First, Fecchi et al. report an updated review focus on lipid rafts, which play pivotal roles in coronavirus entry via endocytosis and in the regulation of autophagy. They discuss the close interplay between lipid rafts and autophagy and reveal that lipid rafts can regulate autophagy by interacting with autophagosomes and autophagy-related proteins. During co-evolution with their natural hosts, viruses have developed the ability to hijack autophagic mechanisms to their advantage by using them for immune evasion or exploiting autophagosomes as a replicative niche. Their review addresses the current knowledge regarding the interaction of coronaviruses with lipid rafts and autophagic pathways, which may be useful for developing novel potential targets to block coronavirus infection.

Second, Sviridov et al. present an ideal solution for developing an antiviral treatment by targeting multiple stages of the viral lifecycle and co-morbidities of SARS-CoV-2 infection. They proposed that lipid rafts are the main sites for the initial binding, internalization, and cell-to-cell transmission of SARS-CoV-2. Most importantly, lipid rafts also play key roles in host immune responses, the dysregulation of which is characteristic of severe COVID-19. The common co-morbidities of COVID-19 including severe inflammation and coagulopathy. Lipid rafts are involved in the regulation of inflammatory response and platelet function, thereby targeting lipid rafts is an effective therapeutic approach to mitigate inflammation and attenuate platelet function. In addition, MβCD can be employed to deliver compounds with limited solubility to cells, such as Remdesivir. However, some agents of lipid raft therapy may possess side effects, which require careful attention. In summary, this review concludes the potential use of “lipid raft therapy” for COVID-19, which has several advantages over other treatment strategies, including (i) the targeting of multiple steps of infection and co-morbidities of COVID-19, (ii) its insensitivity to viral mutation and thus its potential applicability for both the current pandemic and future pandemics, (iii) its safety, broad spectrum activity, and ability to be rapidly repurposed for COVID-19 treatment, and (iv) its potential either as a standalone therapy or in combination with other therapies. Therefore, pharmaceutical agents that reduce cellular cholesterol and thus dissociate membrane rafts are a promising therapeutic strategy to treat emerging viral infections.

As such, this themed collection provides updates on the latest research into the functions of lipid rafts in microbial pathogenesis. Understanding the interactions between microbial pathogens and lipid rafts may provide crucial insights into the mechanisms of infectious diseases and aid in the development of novel therapeutic strategies to prevent or ameliorate the diseases caused by these pathogens.

## Author Contributions

C-HL drafted the manuscript. All authors contributed to manuscript revision, read, and approved the submitted version.

## Conflict of Interest

The authors declare that the research was conducted in the absence of any commercial or financial relationships that could be construed as a potential conflict of interest.

